# Mutation analysis of the *PAH* gene in phenylketonuria patients from Rio de Janeiro, Southeast Brazil

**DOI:** 10.1002/mgg3.408

**Published:** 2018-05-10

**Authors:** Eduardo Vieira Neto, Francisco Laranjeira, Dulce Quelhas, Isaura Ribeiro, Alexandre Seabra, Nicole Mineiro, Lilian d. M. Carvalho, Lúcia Lacerda, Márcia G. Ribeiro

**Affiliations:** ^1^ Gerência de Monitoramento Assistencial Agência Nacional de Saúde Suplementar Rio de Janeiro Brazil; ^2^ Serviço de Genética Médica Instituto de Puericultura e Pediatria Martagão Gesteira Universidade Federal do Rio de Janeiro Rio de Janeiro Brazil; ^3^ Centro de Genética Médica Doutor Jacinto Magalhães Unidade de Bioquímica Genética Porto Portugal; ^4^ Serviço de Metabologia Instituto de Diabetes e Endocrinologia Luiz Capriglione Rio de Janeiro Brazil

**Keywords:** Brazil, epidemiology, haplotypes, mutation analysis, *PAH* gene, phenylketonuria

## Abstract

**Background:**

Phenylketonuria (PKU) is an autosomal recessive disease resulting from mutations in the *PAH* gene. Most of the patients are compound heterozygotes, and genotype is a major factor in determining the phenotypic variability of PKU. More than 1,000 variants have been described in the *PAH* gene. Rio de Janeiro's population has a predominance of Iberian, followed by African and Amerindian ancestries. It is expected that most PKU variants in this Brazilian state have originated in the Iberian Peninsula. However, rare European, African or pathogenic variants that are characteristic of the admixed population of the state might also be found.

**Methods:**

A total of 102 patients were included in this study. Genomic DNA was isolated from dried blood spots. Sanger sequencing was used for PAH gene variant identification. Deletions and duplications were also screened using MLPA analysis. Haplotypes were also determined.

**Results:**

Nine (8.8%) homozygous and 93 (91.2%) compound heterozygous patients were found. The spectrum included 37 causative mutations. Missense, nonsense, and splicing pathogenic variants corresponded to 63.7%, 2.9%, and 22.6% of the mutant alleles, respectively. Large (1.5%), and small deletions, inframe (5.4%) and with frameshift (3.9%), comprised the remainder. The most frequent pathogenic variants were: p.V388M (12.7%), p.R261Q (11.8%), IVS10‐11G>A (10.3%), IVS2+5G>C (6.4%), p.S349P (6.4%), p.R252W (5.4%), p.I65T (4.4%), p.T323del (4.4%), and p.P281L (3.4%). One novel variant was detected: c.934G>T (p.G312C) [rs763115697].

**Conclusion:**

The three most frequent pathogenic variants in our study (34.8% of the alleles) were also the most common in other Brazilian states, Portugal, and Spain (p.V388M, p.R261Q, IVS10‐11G>A), corroborating that the Iberian Peninsula is the major source of PAH mutations in Rio de Janeiro. Pathogenic variants that have other geographical origins, such IVS2+5G>C, p.G352Vfs*48, and IVS12+1G>A were also detected. Genetic drift and founder effect may have also played a role in the mutation spectrum we observed.

## 1 INTRODUCTION

Phenylketonuria (PKU; OMIM # 261600) is a hereditary autosomal recessive disease characterized by an accumulation of the amino acid phenylalanine (Phe) in blood (hyperphenylalaninemia) and other tissues (Donlon, Sarkissian, Levy, & Scriver, 2014). The disease is caused by mutations in the gene encoding the hepatic enzyme phenylalanine hydroxylase (PAH, EC 1.14.16.1), resulting in a decrease or even elimination of enzyme activity, responsible for the conversion of Phe into tyrosine (Tyr), in the presence of its cofactor tetrahydrobiopterin ‐ BH_4_ (van Wegberg et al., 2017). When untreated, the protracted high blood Phe levels cross the blood–brain barrier causing extensive structural damage to the central nervous system (Anderson & Leuzzi, 2010).

The human *PAH* gene (OMIM # 612349) covers approximately 100 kb of genomic DNA, consists of 13 exons and 12 introns, and has been mapped on chromosome 12, band region q23.2 (Donlon et al., 2014). The current number of *PAH* variants in the *PAH*vdb ‐ Phenylalanine Hydroxylase Gene Locus‐Specific Database is 1041 (http://www.biopku.org/pah/search-results-browse.asp), of which circa 630 are disease‐causing mutations, associated with various degrees of PAH deficiency (BIOPKU; http://www.biopku.org/biopku/search-start.asp). These mutations may result in clinical manifestations ranging from mild hyperphenylalaninemia (MHP), which does not require dietary therapy, to a severe and persistent phenotype, classic PKU (Guldberg et al., 1998). Certain mutations are correlated with a specific class of PKU, responsive to tetrahydrobiopterin cofactor ‐ BH_4_ (Trefz, Scheible, Gotz, & Frauendienst‐Egger, 2009).

Approximately 6‐9 different alleles account for the majority of mutant chromosomes described in previous PKU population genetics studies done in Latin America – Brazil (Acosta, Silva, Carvalho, Gomes, & Zago, 2001; Santana da Silva et al., 2003; Santos et al., 2008), Chile (Hamilton et al., 2017), and Mexico (Vela‐Amieva et al., 2015), and the Iberian Peninsula (Aldamiz‐Echevarria et al., 2016; Couce et al., 2013; Rivera et al., 2011); the remainder are minor, rare, and even private disease‐causing alleles.

Additionally, the gene is rich in intragenic polymorphic markers, including seven biallelic restriction‐fragment‐length polymorphisms (RFLP), silent single‐nucleotide polymorphism (SNP) alleles, a short tandem tetranucleotide repeat (STR) in intron 3, and a variable number of tandem repeats (VNTRs) (30‐bp‐length AT‐rich cassettes) in the 3′ untranslated region (Donlon et al., 2014). The polymorphic sites are in linkage disequilibrium and describe a large series of extended and mini‐haplotypes. These haplotypes have been extensively used as tools in population genetics studies to increase the knowledge of the historical and prehistorical movements of human populations, which can explain the contemporary geographic distribution of many *PAH* gene mutant alleles (Rivera et al., 2011).

In the 60s of the 20th century, Guthrie developed a bacterial inhibition test that could detect high amounts of Phe in a dried blood spot (Guthrie & Susi, 1963). This test made it possible to carry out newborn screening test for PKU, enabling early diagnosis and dietary treatment of the disease and the prevention of the development of intellectual disability (van Wegberg et al., 2017). In the next decade, newborn screening programs for PKU were routine in most developed countries.

In Brazil, newborn screening for PKU began in 1976 with the pioneering work of Benjamin Schmidt at a private institution for the intellectually disabled in São Paulo (de Carvalho, dos Santos, dos Santos, Vargas, & Pedrosa, 2007). Only in 2001, the Federal Government implemented the National Newborn Screening Program all over the country (de Carvalho et al., 2007). In Rio de Janeiro, newborn screening started in the 1980s, when the State Institute of Diabetes and Endocrinology (IEDE) and an independent philanthropic organization both put in practice programs for newborn screening of PKU and congenital hypothyroidism, which reached a coverage of 81% of the state's live births in 2007 (Botler, Camacho, & Cruz, 2012). The incidence of PKU in Rio de Janeiro disclosed by this newborn screening program was 1 in 25,000 live births in the same year (Botler et al., 2012). Currently, IEDE is the state reference for the screening, diagnosis and follow‐up of patients with PKU, and circa 150 patients are followed at the institution.

The diversity of mutations in the *PAH* gene found in the Brazilian population seems to be a consequence of genetic drift and founder effect upon a highly admixed population, resulting from five centuries of interaction between three large ethnic groups: Europeans, Africans, and Amerindians (Saloum de Neves Manta et al., 2013). However, the proportion of these three large ethnic groups is very variable in various Brazilian populations, considering that the historical processes of population formation occurred in very diverse ways in each of the regions of the country. To date, four reports have described the molecular basis of PKU in Southeast – São Paulo (Acosta et al., 2001) and Minas Gerais (Santos et al., 2008), and South Brazil (Perez et al., 1996; Santana da Silva et al., 2003). Unpublished material has described PKU mutation spectrum in Northeast Brazil [Boa Sorte, T.R.S.A. (2010) *Estudo de bases moleculares de Fenilcetonúria no Nordeste do Brasil*. (Thesis), Fundação Oswaldo Cruz, Centro de Pesquisas Gonçalo Moniz, Salvador]. The PKU mutational profile in the state of Rio de Janeiro is unknown so far. Rio de Janeiro's population has a predominance of European ancestry ‐ especially Iberian, followed by African and, to a lesser extent, Amerindian ancestry (Manta et al., 2013). It is expected that most of the PKU pathogenic variants in this Southeast Brazil state have originated in the Iberian Peninsula. However, it is likely that mutations that are rare in Europe, of African origin or that are characteristic of the admixed population of the state will also be found. This study seeks to produce knowledge that will contribute to settle these issues.

## 2 MATERIALS AND METHODS

### 2.1 Ethical compliance

This study was approved by the National Research Ethics Commission of Brazil, and conducted in line with the Guidelines and Standards for Research in Human Beings, established by Resolution No. 466/2012 of the Brazilian National Council of Health (Brazil's Ministry of Health. National Council of Health, 2012). Informed consent was obtained from all patients and parents of minors engaged in this study.

### 2.2 Patients

One hundred two patients, including seven pairs of siblings, were included in this study. The patients belonged to 95 unrelated families. The patients sample represents approximately 68% (102/150) of the patients followed at IEDE. The majority of the patients (95) were diagnosed by newborn screening; seven patients had a late diagnosis. A diagnosis of PKU was defined as a blood Phe level ≥10.0 mg/dl (600 μmol/L) in a confirmatory sample. Patients with blood Phe levels persistently ≥6.0–9.9 mg/dl were diagnosed as having MHP. The allocation of patients to one of the following phenotypes was done according to pretreatment Phe levels: classic PKU, ≥1,200 μmol/L (≥20 mg/dl); moderate PKU, ≥900 μmol/L and <1200 μmol/L (≥15 mg/dl and <20 mg/dl); mild PKU, ≥600 μmol/L and <900 μmol/L (≥10 mg/dl and <15 mg/dl); MHP, ≥360 μmol/L and <600 μmol/L (≥6 mg/dl and <10 mg/dl). A total of 54 (52.9%) patients were classified as classic PKU, 25 (24.5%) as moderate PKU, 20 (19.6%) as mild PKU, and 3 (3.0%) as MHP.

### 2.3 Molecular genetic analysis

Dried blood spot samples (DBS) were collected from patients at IEDE in Rio de Janeiro, Brazil, and sent by air transport to the Biochemical Genetics Unity, Dr Jacinto Magalhães Medical Genetics Center, Porto, Portugal, where all genetic analyses were performed. Genomic DNA was isolated from DBS using the EZ1 DNA Tissue Kit in combination with the BioRobot EZ1 workstation (Qiagen, Hilden, Germany). Sanger sequencing was used for *PAH* gene variant identification. Primers for the 13 exons and intronic boundaries of the *PAH* gene were designed employing the NCBI Primer‐BLAST tool (http://www.ncbi.nlm.nih.gov/tools/primer-blast/) (Table S1). These primers were tagged with a M13 sequence for the later cycle sequencing reaction. PCR was carried out using the EmeraldAmp MAX PCR Master Mix (Takara Bio Inc., Kusatsu, Shiga, Japan). PCR products were purified with ExoSAP‐IT (Affymetrix, Santa Clara, CA, USA), and subjected to a cycle sequencing reaction using BigDye Terminators v3.1 kit (Applied Biosystems, Foster City, CA, USA), and M13 primers [M13(‐21)F: 5′‐TGTAAAACGACGGCCAGT‐3′, M13R: 5′‐CAGGAAACAGCTATGACC‐3′]. Sequencing products were purified by gel filtration on a DyeEx 96‐well plate (Qiagen). The purified sequencing products were analyzed in an automatic ABI Prism 3130xl genetic analyzer (Applied Biosystems).

The sequences of all 13 exons and intron‐exon boundaries were aligned and compared with the wild‐type human *PAH* Ensembl reference sequence (ENSG00000171759) [http://www.ensembl.org/Homo_sapiens/Gene/Summary?g=ENSG00000171759;r=12:102836885-102958410] using SeqScape Software v2.5 (Applied Biosystems). The observed variants were referred to the NCBI reference sequence for human *PAH* gDNA, NG_008690.2, and transcript NM_000277.2.

### 2.4 MLPA

After *PAH* gene sequencing, large deletions and duplications were screened by multiplex ligation‐dependent probe amplification (MLPA) in patients in whom only one heterozygous pathogenic variant had been identified. MLPA was performed according to the manufacturer's protocol (MRC‐Holland, Amsterdam, The Netherlands) using the kit SALSA MLPA probemix P055 PAH (Lot # D1‐1015). This contains 20 probes for all the 13 encoding exons of the *PAH* gene (two probes for exons 1‐7 and one probe for exons 8‐13). Between 20 and 50 ng of DNA was used in a MLPA reaction, which was performed on a PCR thermocycler with heated lid (Biometra, Göttingen, Germany). The PCR products were analyzed on an ABI Prism 3100 genetic analyzer (Applied Biosystems) with GeneMapper Software version 4.0 (Applied Biosystems), using as internal standards GeneScan 500 LIZ Size Standards (Applied Biosystems). Data obtained were analyzed by GeneMarker v2.6.0 (Softgenetics, State College, PA, USA).

For normalization, relative probe signals were calculated by dividing each measured peak area by the sum of all peak areas of that sample. Four control samples of healthy individuals were used to calculate the ratio of each relative probe signal from the patient sample. An exon deletion and duplication was considered when the ratio was lower than 0.75 or higher than 1.30, respectively. All positive results were subjected to a second MLPA analysis. To test if a low signal obtained by MLPA was indeed due to a deletion of an exon and not due to a sequence variant in the probes’ target sequences, all the suspected exons were screened for polymorphisms.

### 2.5 Variant pathogenicity criteria

The databases Ensembl Human (*Homo sapiens*) [http://www.ensembl.org/Homo_sapiens/Info/Index], HGMD (http://www.hgmd.cf.ac.uk/ac/index.php) and ClinVar (http://www.ncbi.nlm.nih.gov/clinvar/) were checked in to determine if variants found in our population, especially those in exons or splicing sites, had already been functionally evaluated. The in silico predictive tools PROVEAN, SIFT, PolyPhen‐2, and Mutation Taster were employed to assess the potential biological effect of selected, ambiguously described, rare or new variants ‐ missense, nonsense, and small deletions. Human Splicing Finder was the in silico tool to evaluate some splicing site variants.

### 2.6 Haplotype analysis

Haplotypes were established after PCR of five RFLP biallelic polymorphisms *Bgl*II, *Pvu*IIa, *Pvu*IIb, *Msp*I, and *Xmn*I and of the multiallelic VNTR system at *Hind*III site at the 3′ untranslated region of the *PAH* gene (Rivera, Leandro, Lichter‐Konecki, Tavares de Almeida, & Lechner, 1998). Haplotype numbering was adapted from Eisensmith and Woo (1992), as *Eco*RI and *Eco*RV polymorphisms were not evaluated. To aid in the haplotype determination, certain silent SNPs (c.‐71A>C, IVS2+19T>C, p.Q232Q, p.V245V, and p.L385L) were also examined (Lichter‐Konecki, Schlotter, & Konecki, 1994). Some haplotypes were inferred both from the remaining RFLP and VNTR polymorphisms studied, and the mutation/haplotype association table available at *PAH*db database (http://www.pahdb.mcgill.ca/?Topic=Search&Section=Main&Page=0).

### 2.7 Homozygosity index (*j*) calculation

Homozygosity index (*j*) at the *PAH* locus in a given population is determined by $j=\sum x_{i}^{2}$, where $x_{i}^{2}$ is the frequency of the allele (Aldamiz‐Echevarria et al., 2016; Rivera et al., 2011).

## 3 RESULTS

### 3.1 Mutational spectrum

This study involved the molecular characterization of 204 alleles from 102 patients, including 14 (seven pairs of) siblings. It was possible to complete genotype all the patients. The second mutant allele, a large deletion of exon 5 found in three patients, was detected only by MLPA. Consequently, the diagnostic efficiency of Sanger sequencing plus MLPA reached 100%. The majority of the patients, 93 (91.2%), were compound heterozygous. Nine (8.8%) patients were homozygous ‐ three cases of c.782G>A (p.R261Q), and one case each of c.1162G>A (p.V388M), c.1066‐11G>A (IVS10‐11G>A), c.503delA (p.Y168Sfs*27), c.498C>G (p.Y166*), c.473G>A (p.R158Q), and c.168+5G>C (IVS2+5G>C). Seven of the 9 homozygous patients had a family history of consanguinity.

**Figure 1:**
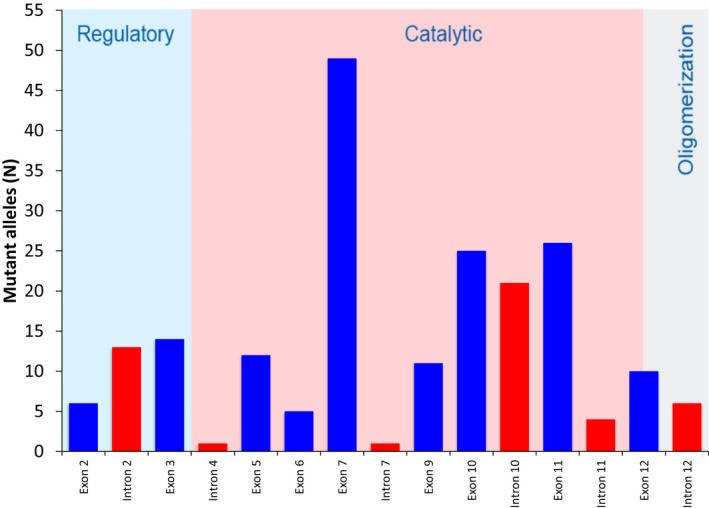
Distribution of *PAH* mutant alleles from 102 PKU/MHP patients from Rio de Janeiro, Brazil, according to gene region and protein domain

The mutational spectrum included 37 causative mutations, which were distributed along almost the entire *PAH* gene sequence (Table 1). Most alleles (67.2%) conveyed mutations that were clustered in exons 7, 11, 10, 3, 5, and 9, with respective frequencies of 24.0%, 12.7%, 12.2%, 6.9%, 5.9%, and 5.4%. A significant number of alleles presented mutations in splicing sites of introns 10 (10.3%) and 2 (6.4%). Exons 12, 2, and 6 carried lower mutant allele frequencies (4.9%, 2.9%, and 2.5%, respectively). The remainder of the alleles displayed mutations in splicing sites of introns 12 (2.9%), 11 (2.0%), 4 (0.5%), and 7 (0.5%). No causative mutations were identified in exons 1, 4, 8, and 13. The 37 causative mutations were distributed in this manner: 22 in the catalytic domain (59.5%), seven in the regulatory domain (18.9%), two in the tetramerization domain (5.4%) and six in the intronic regions (16.2%). Figure 1 summarizes data on the distribution of the mutant alleles according to gene region and protein domain.

**Table 1:** Mutational spectrum found in 102 phenylketonuria (PKU)/mild hyperphenylalaninemia (MHP) patients from Rio de Janeiro, Southeast Brazil

DNA change^a^	Protein effect or trivial name	Type	Gene region	Protein domain	*PAH*vdb^b^	N	RF^b^ (%)	N/RF‐UI^c^ (%)
c.1162G>A	p.V388M	Missense	Exon 11	Catalytic	Deleterious	26	12.7	22/11.6
c.782G>A	p.R261Q	Missense	Exon 7	Catalytic	Deleterious	24	11.8	23/12.1
c.1066‐11G>A	p.Q355_Y356insGLQ^e^	Splicing	Intron 10	—	Not defined^h^	21	10.3	21/11.0
c.168+5G>C	IVS2+5G>C	Splicing	Intron 2	—	Not defined^i^	13	6.4	13/6.8
c.1045T>C	p.S349P	Missense	Exon 10	Catalytic	Deleterious	13	6.4	11/5.8
c.754C>T	p.R252W	Missense	Exon 7	Catalytic	Deleterious	11	5.4	10/5.3
c.194T>C	p.I65T	Missense	Exon 3	Regulatory	Deleterious	9	4.4	9/4.7
c.967_969delACA	p.T323del	Inframe deletion	Exon 9	Catalytic	Not defined^h^	9	4.4	8/4.2
c.842C>T	p.P281L	Missense	Exon 7	Catalytic	Deleterious	7	3.4	7/3.7
c.1042C>G	p.L348V	Missense	Exon 10	Catalytic	Deleterious	6	2.9	5/2.6
c.1315+1G>A	IVS12+1G>A	Splicing	Intron 12	—	Not defined^h^	6	2.9	5/2.6
c.473G>A	p.R158Q	Missense	Exon 5	Catalytic	Deleterious	5	2.5	5/2.6
c.1055delG	p.G352Vfs*48	Frameshift deletion	Exon 10	Catalytic	Not defined^h^	5	2.5	5/2.6
c.1241A>G	p.Y414C	Missense	Exon 12	Oligomerization	Deleterious	5	2.5	5/2.6
c.745C>T	p.L249F	Missense	Exon 7	Catalytic	Deleterious	4	2.0	4/2.1
c.1199+17G>A	IVS11+17G>A	Splicing	Intron 11	—	Not defined^i^	4	2.0	4/2.1
c.250G>T	p.D84Y	Missense	Exon 3	Regulatory	Deleterious	3	1.5	3/1.6
c.442‐?_509+?del	Unknown	Large deletion	Exon 5	Catalytic	Not defined^l^	3	1.5	3/1.6
c.116_118delTCT	p.F39del	Inframe deletion	Exon 2	Regulatory	Not defined^j^	2^g^	1.0	1/0.5
c.136G>A	p.G46S	Missense	Exon 2	Regulatory	Deleterious	2	1.0	2/1.1
c.204A>T	p.R68S	Missense	Exon 3	Regulatory	Deleterious	2	1.0	2/1.1
c.498C>G	p.Y166*	Nonsense	Exon 5	Catalytic	Not defined^h^	2^f^	1.0	2/1.1
c.503delA	p.Y168Sfs*27	Frameshift deletion	Exon 5	Catalytic	Not defined^h^	2^f^	1.0	2/1.1
c.526C>T	p.R176*	Nonsense	Exon 6	Catalytic	Not defined^h^	2	1.0	2/1.1
c.561G>C	p.W187C	Missense	Exon 6	Catalytic	Deleterious	2^g^	1.0	1/0.5
c.809G>A	p.R270K	Missense	Exon 7	Catalytic	Deleterious	2	1.0	2/1.1
c.934G>T^d^	p.G312C	Missense	Exon 9	Catalytic	—	2^g^	1.0	1/0.5
c.1222C>T	p.R408W	Missense	Exon 12	Catalytic	Deleterious	2	1.0	2/1.1
c.1243G>A	p.D415N	Missense	Exon 12	Oligomerization	Tolerated	2	1.0	2/1.1
c.143T>C	p.L48S	Missense	Exon 2	Regulatory	Deleterious	1	0.5	1/0.5
c.165delT	p.F55Lfs*6	Frameshift deletion	Exon 2	Regulatory	Not defined^j^	1	0.5	1/0.5
c.441+5G>T	IVS4+5G>T	Splicing	Intron 4	—	Not defined^j^	1	0.5	1/0.5
c.618C>G	p.Y206*	Nonsense	Exon 6	Catalytic	Not defined^i^	1	0.5	1/0.5
c.781C>T	p.R261*	Nonsense	Exon 7	Catalytic	Not defined^h^	1	0.5	1/0.5
c.842+1G>A	IVS7+1G>A	Splicing	Intron 7	—	Not defined^j^	1	0.5	1/0.5
c.994G>A	p.G332R	Missense	Exon 10	Catalytic	Not defined^k^	1	0.5	1/0.5
c.1223G>A	p.R408Q	Missense	Exon 12	Catalytic	Deleterious	1	0.5	1/0.5
Total						204	100	190/100

*Stop codon.

a Reference sequence: NM_000277.2.

Pathogenicity according to *PAH*vdb ‐ Phenylalanine Hydroxylase Gene Locus‐Specific Database (http://www.biopku.org/home/pah.asp) ‐ SIFT interpretation.

b RF: relative frequency.

c RF‐UI: relative frequency in unrelated individuals.

d Novel mutation, dbSNP: 763115697.

e Trivial name: IVS10‐11G>A.

f One homozygous patient.

g Two siblings.

h Reported to ClinVar as pathogenic.

i Reported to ClinVar as likely pathogenic.

j Reported to ClinVar as pathogenic/likely pathogenic.

k Not previously reported to Clinvar.

l Pathogenicity not informed by ClinVar either.

Most mutant alleles carried nucleotide substitutions, of which 130 were missense mutations (63.7%), 46 mutations at splicing sites (22.6%), and 6 nonsense mutations (2.9%). Large (three mutant alleles ‐ 1.5%), and small deletions, inframe (11 mutant alleles ‐ 5.4%) and with frameshift (eight mutant alleles ‐ 3.9%), comprised the remainder.

**Figure 2:**
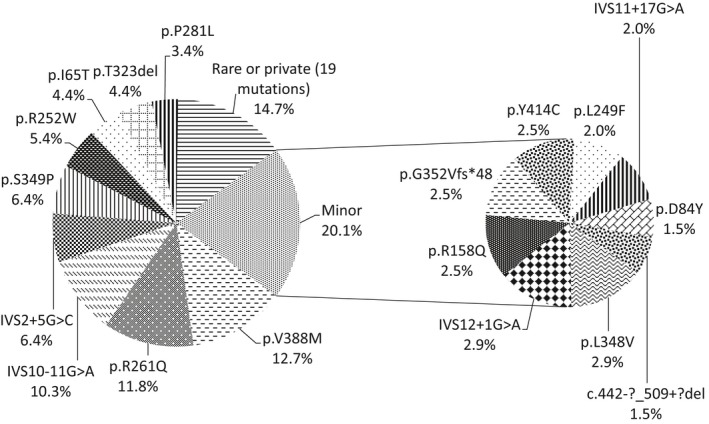
Relative frequencies of *PAH* mutant alleles from 102 PKU/MHP patients from Rio de Janeiro, Brazil

Thirteen causative mutations were observed in only one patient or in two related individuals (private mutations), and six mutations were found in only two unrelated individuals, totaling 19 rare mutations (frequency <1.5%) (Table 1; Figure 2). The most prevalent pathogenic variant in our population was c.1162G>A (p.V388M), in exon 11, found in 26 mutant alleles (12.7%). Four pairs of siblings were compound heterozygotes for this variant; therefore, it was the second most frequent variant (11.6% ‐ 22 in 190) among mutant alleles in unrelated individuals. The next two most frequent pathogenic variants were c.782G>A (p.R261Q), in exon 7, and c.1066‐11G>A (IVS10‐11G>A), in intron 10, which displayed a relative frequency of 11.8%, and 10.3% of the mutant alleles, respectively. The relative frequencies of these two variants in mutant alleles of unrelated patients were 12.1% (23 in 190 ‐ highest frequency) and 11.0% (21 in 190), respectively (Table 1). The three most common pathogenic variants accounted for 34.8% of the mutant alleles. Six other major variants (present in >3% of the mutant alleles) were found: c.168+5G>C (IVS2+5G>C) [13 alleles; 6.4%], c.1045T>C (p.S349P) [13; 6.4%], c.754C>T (p.R252W) [11; 5.4%], c.194T>C (p.I65T) [9; 4.4%], c.967_969delACA (p.T323del) [9; 4.4%], and c.842C>T (p.P281L) [7; 3.4%] (Table 1; Figure 2). Eight minor variants (present in ≥1.5%‐<3% of the mutant alleles) were as follows: c.1042C>G (p.L348V) [6 alleles; 2.9%], c.1315+1G>A (IVS12+1G>A) [6; 2.9%], c.473G>A (p.R158Q) [5; 2.5%], c.1055delG (p.G352Vfs*48) [5; 2.5%], c.1241A>G (p.Y414C) [5; 2.5%], c.745C>T (p.L249F) [4; 2.0%], c.1199+17G>A (IVS11+17G>A) [4; 2.0%], c.250G>T (p.D84Y) [3; 1.5%], and c.442‐?_509+?del, a large deletion of exon 5, with uncharacterized breakpoints [3; 1.5%] (Table 1; Figure 2).

**Figure 3:**
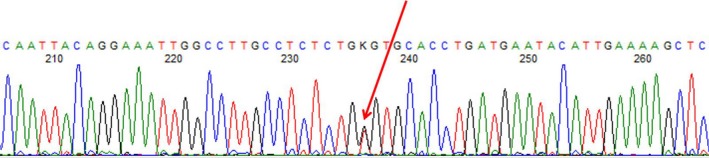
Sanger sequencing electropherogram of a PKU patient sample showing the novel variant c.934G>T (p.G312C), a missense variant in the catalytic domain of exon 9, in the compound heterozygous state

One novel variant was detected: c.934G>T (p.G312C) [rs763115697], accession number PAH1068 in *PAH*vdb, a missense variant in exon 9, affecting residue 312 in the protein catalytic domain (Figure 3). It was found out in two early‐treated white sisters with a moderate/mild form of PKU, in compound heterozygous with IVS12+1G>A (c.1315+1G>A), a splicing variant in intron 12. The in silico analysis of this variant employing the tools SIFT (http://sift.jcvi.org/www/SIFT_enst_submit.html), Provean (http://provean.jcvi.org/seq_submit.php), and PolyPhen‐2 (http://genetics.bwh.harvard.edu/pph2/) revealed scores of 0 (damaging), −8.734 (deleterious), and 1.00 (probably damaging), respectively. Mutation Taster (http://www.mutationtaster.org) predicted the variant to be disease causing with a probability >.9999. Using a computational algorithm to specific address this variant potential effect on splicing, Human Splicing Finder (http://www.umd.be/HSF3) revealed a potential alteration of splicing, an activation of an exonic cryptic donor site, and the creation of an exonic splicing silencer (ESS) site.

Another missense variant c.934G>A (p.G312S) is also described at dbSNP database at rs763115697 (https://www.ncbi.nlm.nih.gov/snp/rs763115697#gene_change_pr_22_tp), however it is clearly distinct from c.934G>T, the novel variant that is being described in this paper. Moreover c.934G>A, contrary to our novel variant (https://www.ncbi.nlm.nih.gov/clinvar/variation/446506/), has not been reported to ClinVar or *PAH*vdb (http://www.biopku.org/pah/search-start.asp). The variant c.934G>C (p.G312R) described from PKU families in India (Bashyam et al., 2014), and reported at *PAH*vdb, but not at dbSNP, although affecting the same transcript position as our novel mutation, has a completely different effect on amino acid sequence (p.G312R).

Applying the criteria for classifying a variant as pathogenic recommended by the American College of Medical Genetics and Genomics and the Association for Molecular Pathology (Richards et al., 2015), we arrived at the conclusion that the new variant c.934G>T (p.G312C) is likely pathogenic. Two moderate pathogenic criteria were identified – PM1 (hot‐spot region with 10 pathogenic variants out of 10 classified variants, 100.0%, which is >70.0%, using a hot‐spot width of 36 base pairs), and PM2 (allele not found in Exome Sequencing Project, 1000 Genomes Project, and Exome Aggregation Consortium). In addition, three supporting criteria were also identified – PP1 (cosegregation with PKU in two sisters in gene *PAH*, definitively known to cause the disease), PP2 (missense variant in gene *PAH* that has 317 pathogenic missense variants versus 1 benign missense variant, ratio 317.0, which is greater than the threshold three, associated with PKU and mild hyperphenylalaninemia), and PP3 (five pathogenic predictions from SIFT, Provean, Poly‐Phen‐2, MutationTaster, and Human Splicing Finder vs. no benign predictions).

Homozygosity rate in Rio de Janeiro PKU population revealed a high genetic heterogeneity (*j *= 0.063), similar to that found in South Portugal (0.059) (Rivera et al., 2011), Galicia (0.054) (Couce et al., 2013), USA (nationwide index of 0.06) (Guldberg et al., 1996), and in California for the European descent subgroup (0.06) (Enns et al., 1999). Nevertheless, considering that the higher the value of *j*, the more homogeneous the population with respect to the *PAH* locus, our population was more homogeneous than the PKU population of Spain (0.029) (Aldamiz‐Echevarria et al., 2016), and the Hispanic population of California (0.04) (Enns et al., 1999).

### 3.2 Haplotype characterization and association with mutant alleles

Of the 204 mutant alleles ascertained in this study, it was possible to resolve completely 188 (92.2%) of the associated haplotypes. VNTR was settled on for all mutant chromosomes. The lack of *Eco*RV and *Eco*RI RFLP evaluation did not permit to determine precisely some haplotypes linked with mutant alleles: 5.9/6.9 haplotypes associated with two c.116_118delTCT (p.F39del) mutant chromosomes, and one c.994G>A (p.G332R) mutant chromosome; 39.7/60.7 associated with one IVS10‐11G>A mutant chromosome; 14.8/15.8 associated with one c.498C>G (p.Y166*) mutant chromosome, the novel c.934G>T (p.G312C) variant (two alleles in two siblings), and with two out of three mutant chromosomes bearing the large exon 5 deletion, c.442‐?_509+?del, detected by MLPA (for the third chromosome, also on VNTR 8, we could not predict the haplotype based on RFLP results); 2.3/24.3 associated with three p.R261Q mutant chromosomes – the other 2.3 linkage disequilibria were settled on according to Rivera et al. (2011) and Zschocke and Hoffmann (1999); three other alleles associated each with VNTR3, VNTR7, and VNTR9 (Table 2). The analysis revealed that approximately 16 different haplotypes were associated with the mutant alleles (Table 2). Haplotype 1 was the most prevalent among mutant alleles with 16 different mutations associated with it, 14 with VNTR 8 (haplotype 1.8), and four with VNTR 7 (haplotype 1.7). The other completely resolved haplotypes in mutant chromosomes were associated with only one VNTR: 2.3, 3.8, 4.3, 5.9, 6.7, 7.8, 9.8, 11.8, 15.9, and 52.8. Among these, haplotypes 6.7, 11.8, 15.9, and 52.8 were associated with only one pathogenic variant, while the others were linked with 2–4 mutations. Eight of the nine most frequent mutations (>3.0% of the mutant alleles), p.V388M, p.R261Q, IVS10‐11G>A, p.S349P, p.R252W, p.I65T, p.T323del, and p.P281L were in linkage disequilibria to the haplotype usually associated to them in the Iberian Peninsula, especially Portugal: 1.7, 1.8, 6.7, 4.3, 1.8, 9.8, 1.8, and 2.3/1.8 respectively (Table 2). The pathogenic variant IVS2+5G>C, of non‐Iberian origin, was associated to haplotype 5.9, as in other states of Brazil (Acosta et al., 2001; Santos et al., 2008), Germany and Turkey (Zschocke & Hoffmann, 1999). There were also important exceptions to the aforementioned linkages: the association of p.V388M to haplotype 1.8 in two patients, an occurrence previously observed in Minas Gerais, Brazil (Santos et al., 2008) and Spain (Perez, Desviat, & Ugarte, 1997); the linkage of p.R261Q with haplotype 2.3/24.3 in three patients, also formerly reported in Minas Gerais (Santos et al., 2008) and São Paulo, Brazil (Acosta et al., 2001); the finding of the rare haplotype 39.7/60.7 in a patient carrying the IVS10‐11G>A pathogenic variant, previously reported for a homozygous patient in São Paulo (Acosta et al., 2001; Perez et al., 1996); and the association of p.R252W to haplotype 52.8 in one mutant allele, formerly found in two alleles in São Paulo (Acosta et al., 2001).

**Table 2:** *PAH* gene mutations and linked haplotypes in phenylketonuria (PKU)/ mild hyperphenylalaninemia (MHP) patients from Rio de Janeiro, Southeast Brazil

DNA change^a^	Protein effect or trivial name	*N*	Relative frequency (%)	Haplotype (N)^b^	Previous linkage description
c.1162G>A	p.V388M	26	12.7	1.7 (24)	MG, SP, Ch, Cu, P, E^e^
				1.8 (2)	MG, E
c.782G>A	p.R261Q	24	11.8	1.8 (21)	MG, SP, Ch, P, E
				X.3 (3)^f^	MG, SP
c.1066‐11G>A	p.Q355_Y356insGLQ^d^	21	10.3	6.7 (20)	MG, SP, Ch, Cu, P, E
				X.7 (1)^g^	SP
c.168+5G>C	IVS2+5G>C	13	6.4	5.9 (12)	MG, SP
				X.9 (1)^h^	—
c.1045T>C	p.S349P	13	6.4	4.3 (13)	P
c.754C>T	p.R252W	11	5.4	1.8 (9)	MG, SP, P
				1.7 (1)	—
				52.8 (1)	SP
c.194T>C	p.I65T	9	4.4	9.8 (9)	MG, SP, Ch, P, E
c.967_969delACA	p.T323del	9	4.4	1.8 (9)	P
c.842C>T	p.P281L	7	3.4	2.3 (4)^f^	SP, P
				1.8 (3)	MG, SP, P, E
c.1042C>G	p.L348V	6	2.9	9.8 (6)	MG, SP, P
c.1315+1G>A	IVS12+1G>A	6	2.9	3.8 (6)	MG, SP, Ch
c.473G>A	p.R158Q	5	2.5	4.3 (5)	MG, SP, Cu, P
c.1055delG	p.G352Vfs*48	5	2.5	2.3 (5)^f^	MG, SP
c.1241A>G	p.Y414C	5	2.5	4.3 (5)	SP, Ch, P, E
c.745C>T	p.L249F	4	2.0	1.7 (4)	MG
c.1199+17G>A	IVS11+17G>A	4	2.0	7.8 (4)	SP
c.250G>T	p.D84Y	3	1.5	15.9 (2)	—
				X.3 (1)^n^	—
c.442‐?_509+?del	Unknown	3	1.5	X.8 (2)^j^	—k
				X.8 (1)^m^	—k
c.116_118delTCT	p.F39del	2	1.0	X.9 (2)^i^	SP
c.136G>A	p.G46S	2	1.0	5.9 (2)^i^	—k
c.204A>T	p.R68S	2	1.0	1.8 (2)	SP, P, E
c.498C>G	p.Y166*	2	1.0	1.8 (1)	—l
				X.8 (1)^j^	—
c.503delA	p.Y168Sfs*27	2	1.0	3.8 (2)	—l
c.526C>T	p.R176*	2	1.0	1.8 (1)	MG, SP, P
				7.8 (1)	—
c.561G>C	p.W187C	2	1.0	1.8 (2)	—l
c.809G>A	p.R270K	2	1.0	1.8 (2)	SP, P
c.934G>T^c^	p.G312C	2	1.0	X.8 (2)^j^	—
c.1222C>T	p.R408W	2	1.0	2.3 (2)^f^	MG, SP, Ch, Cu
c.1243G>A	p.D415N	2	1.0	7.8 (2)	E
c.143T>C	p.L48S	1	0.5	4.3 (1)	MG, SP, E
c.165delT	p.F55Lfs*6	1	0.5	11.8 (1)	—
c.441+5G>T	IVS4+5G>T	1	0.5	1.8 (1)	—k
c.618C>G	p.Y206*	1	0.5	1.8 (1)	—k
c.781C>T	p.R261*	1	0.5	1.8 (1)	MG, SP
c.842+1G>A	IVS7+1G>A	1	0.5	1.8 (1)	Ch, E
c.994G>A	p.G332R	1	0.5	X.9 (1)^i^	—k
c.1223G>A	p.R408Q	1	0.5	X.7 (1)	—
Total		204	100	—	—

*Stop codon.

a Reference sequence: NM_000277.2.

b Haplotype numbering corresponds to that of Eisensmith and Woo (1992), which includes RFLP polymorphisms and VNTR. An “X” denotes RFLP haplotype uncertainty due to the lack of *Eco*RI and *Eco*RV polymorphism assays.

c Novel mutation, dbSNP: 763115697.

d Trivial name: IVS10‐11G>A.

e Data were quoted from Santos et al. (2008), in Minas Gerais, Brazil (MG), Acosta et al. (2001, 2001), in São Paulo, Brazil (SP), Perez et al. (1999), in Chile (Ch), Desviat et al. (2001), in Cuba (Cu), Rivera et al., 2011; in Portugal (P), and Perez et al., 1997, in Spain (E).

f Haplotype 2.3 cannot be distinguished from 24.3 without *Eco*RV RFLP assay; for p.P281L, p.G352Vfs*48, and p.R408W settled on as 2.3 according to Rivera et al. (2011), for the first, and Zschocke and Hoffmann (1999), for the last two.

g Haplotype 39.7 or 60.7: *Eco*RI RFLP assay capable of distinguishing them not done.

h RFLP haplotype tentatively settled on as 77 (*Bgl*II +, *Pvu*II(a) +, *Pvu*II(b) +, *Msp*I +, *Xmn*I –).

i Haplotype 5.9 or 6.9: *Eco*RV RFLP assay capable of distinguishing them not done; for p.G46S settled on as 5.9 according to Zschocke and Hoffmann (1999) and the mutation/haplotype association table available at PAHdb database (http://www.pahdb.mcgill.ca/?Topic=Search&Section=Main&Page=0).

j Haplotype 14.8 or 15.8: *Eco*RV RFLP assay capable of distinguishing them not done.

k Mutation previously described in Chile (Hamilton et al., 2017), Mexico (Vela‐Amieva et al., 2015), Spain (Aldamiz‐Echevarria et al., 2016; Trujillano et al., 2014) and/or Galicia (Couce et al., 2013) but haplotype not informed.

l Mutation not previously described in Brazilian, Latin American, and Iberian Peninsula populations.

m Undetermined haplotype, not 14.8 or 15.8.

n RFLP haplotype tentatively settled on as 69 (*Bgl*II +, *Pvu*II(a) –, *Pvu*II(b) –, *Msp*I +, *Xmn*I –).

## 4 DISCUSSION

**Figure 4:**
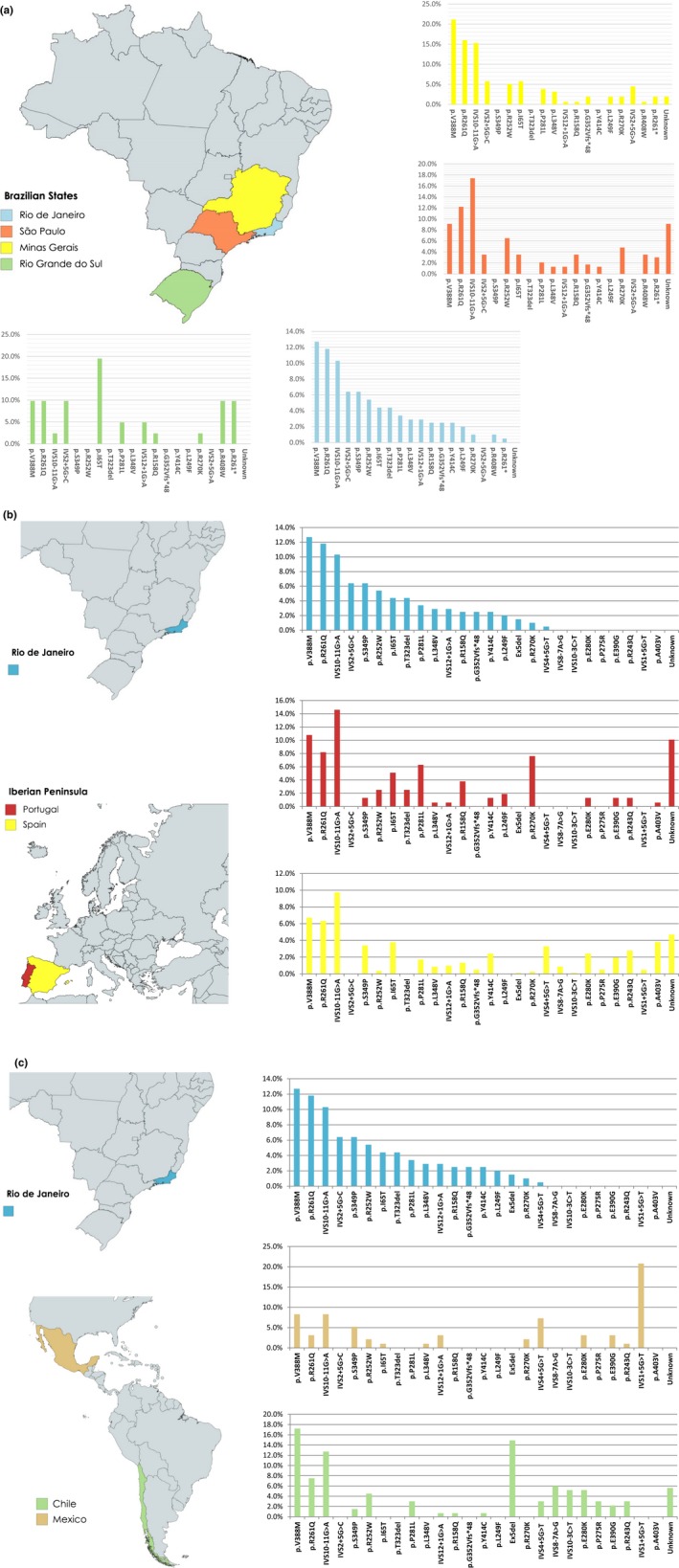
(a) Relative frequencies of prevalent *PAH* mutant alleles in different Brazilian regions. These data were quoted from the present study in Rio de Janeiro, Santos et al. (2008), in Minas Gerais, Acosta et al. (2001), in São Paulo, and Santana da Silva et al. (2003), in South Brazil (Rio Grande do Sul). (b) Relative frequencies of prevalent *PAH* mutant alleles in Rio de Janeiro, Brazil, and the Iberian Peninsula. These data were quoted from the present study in Rio de Janeiro, Rivera et al. (2011), in Portugal, and Aldamiz‐Echevarria et al. (2016), in Spain. (c) Relative frequencies of prevalent *PAH* mutant alleles in Rio de Janeiro, Brazil, and Hispanic America. These data were quoted from the present study in Rio de Janeiro, Hamilton et al. (2017), in Chile, and Vela‐Amieva et al. (2015), in Mexico

The three most frequent mutations in our study, representing 34.8% of the alleles, were also the most common in Southeast Brazil (Acosta et al., 2001; Santos et al., 2008), Portugal (Rivera et al., 2011), and Spain (Aldamiz‐Echevarria et al., 2016): p.V388M, p.R261Q, IVS10‐11G>A. Nevertheless, in Spain they represented only 22.7% of the PKU alleles (*p *=* *.0004), and in Minas Gerais they responded for 52.5% of the alleles (*p *=* *.0008). These differences may reflect the higher heterogeneity of PKU alleles in Spain (*j *=* *0.029), and the lower heterogeneity in Minas Gerais, Southeast Brazil (*j *=* *0.110), in comparison to our population (Figure 4a,b). In South Brazil, IVS10‐11G>A had a much lower frequency among PKU alleles than in Southeast Brazil (Figure 4a), the Iberian Peninsula (Figure 4b), and Hispanic America (Figure 4c). The low number of PKU alleles analyzed from South Brazil patients [41] (Santana da Silva et al., 2003) precludes any further conclusion concerning the observed differences.

We could not find in our population p.V388M PKU alleles on haplotype 4.3, an association of probable Amerindian inheritance found in Chile and Mexico (Desviat et al., 1995).

Two other frequent mutations of probable Iberian origin, p.S349P (6.4%) and p.T323del (4.4%), associated with haplotypes 4.3 and 1.8, respectively, have not been previously described in Brazil (Acosta et al., 2001; Santana da Silva et al., 2003; Santos et al., 2008). The missense pathogenic variant, p.S349P, has been observed in 3.4%, 1.3%, and 1.0% of PKU alleles in Spain (Aldamiz‐Echevarria et al., 2016), South Portugal (Rivera et al., 2011), and Galicia (Couce et al., 2013), respectively. In South Portuguese alleles it was also found on haplotype 4.3; we are not aware of any haplotype linkage data for Spanish and Galician p.S349P alleles. In turn, the inframe deletion, c.967_969delACA (p.T323del), has been observed in 2.5% of South Portuguese alleles, on haplotypes 1.7 and 1.8. The variant p.S349P on haplotype 4.3 was firstly observed in PKU alleles in Jews from Morocco and Tunisia (Weinstein et al., 1993); c.967_969delACA (p.T323del) was originally described in one mutant allele from a Californian patient of Armenian heritage (Enns et al., 1999). The higher frequency of these two mutations in comparison to the Iberian Peninsula alleles, yet on the same haplotypes as in South Portugal, are evidence for the important roles of genetic drift and founder effect in shaping the mutational spectrum of PKU in Rio de Janeiro.

Other major mutations (>3.0% of mutant alleles) of probable Iberian origin were c.754C>T (p.R252W), c.194T>C (p.I65T), and c.842C>T (p.P281L) (Figure 2). The frequencies of these mutations among PKU alleles in the three Southeast Brazil states and in Portugal were of the same order of magnitude (Figure 4a,b), while p.R252W was relatively rare (0.4%) among PKU alleles in Spain (Aldamiz‐Echevarria et al., 2016). Alleles carrying p.R252W were mainly on haplotype 1.8 in our population, supporting the Portuguese origin of this pathogenic variant, since in other European populations it is found on different haplotypes, for example, 7.8 in Central Europe (Zschocke & Hoffmann, 1999; Zschocke et al., 2003), in individuals of confirmed or potential Central European ancestry in Chile (Perez et al., 1999), and Australia (Ramus, Treacy, & Cotton, 1995); and X.3 in Spain (Perez et al., 1997).

A total of 15 of the 48 mutant allele‐linked haplotype combinations found in our PKU population, comprising 133 of the 204 mutant alleles (65.2%), was probably of Portuguese origin.

The most frequent pathogenic *PAH* variant of non‐Iberian origin, IVS2+5G>C (6.4%), associated with haplotype 5.9, has been previously reported as a common variant in Southeast and South Brazil (Figure 4a). It has not been reported in Portugal, Spain, and Hispanic America (Desviat et al., 2001; Hamilton et al., 2017; Perez et al., 1999; Vela‐Amieva et al., 2015) (Figure 4b,c). It is found in Middle Eastern and Central and Eastern European PKU patients (Biglari et al., 2015; Danecka et al., 2015; Kasnauskiene, Giannattasio, Lattanzio, Cimbalistiene, & Kucinskas, 2003; Zschocke & Hoffmann, 1999; Zschocke et al., 2003).

The second most frequent pathogenic variant of non‐Iberian origin was c.1315+1G>A (IVS12+1G>A), on haplotype 3.8, a splicing pathogenic variant of North European origin. Rare in Portugal (0.6%) and Spain (1.0%), it reached a reasonable frequency in our population, 2.9% of PKU alleles (statistically higher than in Spain's PKU population – *p *=* *.0327, but not Portugal's – *p *=* *.1175).

An interesting contribution of non‐Iberian European heritage to the PKU allele diversity in our population was the pathogenic variant c.136G>A (p.G46S). It was found in two PKU alleles of two compound heterozygous unrelated males (one of mixed, African and European, ethnicity), both linked with haplotype 5.9. The variant has been reported in 2.4% and 1.0% of Galician (Couce et al., 2013) and Spanish (Aldamiz‐Echevarria et al., 2016) PKU alleles, respectively. But it is in Scandinavia that it reaches its peak, being present in 7.2% of Norwegian PKU alleles (Eiken et al., 1996) and in 4.3% of Swedish PKU alleles (Ohlsson et al., 2017), also linked with haplotype 5.9.

Some rare *PAH* pathogenic variants, found previously in geographical areas with no historical links to Rio de Janeiro, may represent recurrent mutational events. They have not been previously described in other Brazilian regions (Acosta et al., 2001; Santana da Silva et al., 2003; Santos et al., 2008), Portugal (Rivera et al., 2011) and Spain (Aldamiz‐Echevarria et al., 2016; Couce et al., 2013; Perez et al., 1997; Trujillano et al., 2014). The nonsense pathogenic variant c.498C>G (p.Y166*) was observed in a homozygous early‐treated black female, with no history of consanguinity, being each mutant allele linked with a distinct haplotype: 1.8 and 14.8/15.8. This variant was originally described in Southwestern China (Wang et al., 1999), and has been subsequently found in other parts of China (Song et al., 2005; Yu et al., 2014). The frameshift deletion c.503delA (p.Y168 fs*27) was detected in a homozygous early‐treated white female, child of a consanguineous couple, linked with haplotype 3.8. It has been formerly reported in a heterozygous patient from Australia (Ho et al., 2014), and in two patients by a private reference laboratory in Georgia, USA, reported to ClinVar. Finally, the missense variant c.561G>C (p.W187C), observed in our population in two early‐treated white heterozygous siblings, was reported for the first time in a PKU patient in Japan (Okano et al., 1998), and, as far as we know, has not been described in other populations. In these siblings, it was linked with haplotype 1.8.

Other rare mutations might be of Spanish or Hispanic origin as, although not previously described in Brazil and Portugal, they have indeed been reported in Spain, Galicia or Hispanic America. The splicing variant c.441+5G>T (IVS4+5G>T) was found in a patient whose other allele carried a large deletion of exon 5 (c.442‐?_509+?del). This pathogenic variant is frequent in Mexico and Chile, representing 7.3%, and 3.0% of the mutant alleles in PKU patients in those countries, respectively (Hamilton et al., 2017; Vela‐Amieva et al., 2015). The nonsense pathogenic variant c.618C>G (p.Y206*) was originally described on haplotype 1 (VNTR not determined) in a patient of Spanish ethnicity in Belgium (Michiels, Francois, Raus, & Vandevyver, 1998), see also the *PAH*db database (http://www.pahdb.mcgill.ca/?Topic=Search&Section=Main&Page=0). Not previously described in Brazil or Portugal, the variant occurs in 0.2% of Spanish alleles (Aldamiz‐Echevarria et al., 2016). It was observed in a late diagnosed compound heterozygous white male on haplotype 1.8. The missense pathogenic variant c.994G>A (p.G332R) was firstly described in a compound heterozygous Spanish patient, with a large deletion of exon 5 on the other allele (Trujillano et al., 2014). It was detected in a early‐treated compound heterozygous male, on haplotype X.9 (5.9 or 6.9), with c.1162G>A (p.V388M) on the other allele.

The geographic or ethnic origin of some rare mutant alleles was hard to resolve. The c.250G>T (p.D84Y) is an striking example of this uncertainty. Two of these alleles were found on haplotype 15.9. A third allele was found on VNTR 3, haplotype tentatively settled on as 69. These alleles were from three unrelated early‐treated compound heterozygous males, two black and the third white. In the Czech Republic and in Germany, c.250G>T (p.D84Y) alleles have been reported on 4.3 haplotype, according to the *PAH*db database (http://www.pahdb.mcgill.ca/?Topic=Search&Section=Main&Page=0). In Minas Gerais, Brazil, two c.250G>T (p.D84Y) alleles were previously described on haplotypes 5.9 and 11.9 (Santos et al., 2008). Haplotype 15.9 differs from haplotypes 11.9 and 5.9 by one and two point mutations at RFLP sites, respectively. In turn, haplotypes 4.3 and 69.3 differ by four point mutations. Consequently, the c.250G>T (p.D84Y) allele on VNTR 3 found in a black male in our study probably does not share a common origin with Czech and German alleles, and the relationship among the Brazilian c.250G>T (p.D84Y) alleles on haplotypes 15.9, 11.9, and 5.9 remains to be settled. The two c.526C>T (p.R176*) alleles found in our population might have different geographic origins. One was found on haplotype 7.8 in an early‐treated compound heterozygous mestizo (black and white) male. Another allele, in an early‐treated compound heterozygous white female, was detected on the usual 1.8 haplotype as formerly reported in São Paulo (Acosta et al., 2001), Minas Gerais (Santos et al., 2008), and Portugal (Rivera et al., 2011). Haplotypes 7.8 and 1.8 differ by five point mutations at RFLP sites, as a result, these two alleles probably do not share a common origin. Finishing the list of rare variants of uncertain origin, a c.165delT (p.F55Lfs*6) allele was observed on haplotype 11.8 in an early‐treated compound heterozygous male. One c.165delT (p.F55Lfs*6) allele was formerly detected on haplotype 1.8 in Minas Gerais (Santos et al., 2008), the same haplotype on which alleles from the United Kingdom, Germany, Norway, and Italy were reported, according to the *PAH*db database (http://www.pahdb.mcgill.ca/?Topic=Search&Section=Main&Page=0). We can deduce that c.165delT (p.F55Lfs*6) 1.8 and c.165delT (p.F55Lfs*6) 11.8 do not share a common origin as haplotypes 11.8 and 1.8 differ by four point mutations at RFLP sites. The variant c.165delT (p.F55Lfs*6) on haplotype 1.8 might represent a recurrent mutational event.

It is worth turning our attention to the large deletion covering exon 5 (c.442‐?_509+?del) found in three mutant alleles exclusively by MLPA. Large deletions in *PAH* gene have not been previously detected among PKU alleles in Brazil (Acosta et al., 2001; Santana da Silva et al., 2003; Santos et al., 2008). However, this is probably not due to its absence in the Brazilian PKU population, but a result of the masking effect of the non‐deleted allele that do not permit the identification of deletions encompassing one or more whole exons in a heterozygous state, when standard variant screening methods such as Sanger sequencing are exclusively employed (Birk Moller et al., 2007; Cali et al., 2010). Large deletions of exon 5 have been formerly reported, among other countries, in Chile (Hamilton et al., 2017), Spain (Desviat, Perez, & Ugarte, 2006; Trujillano et al., 2014), Italy (Cali et al., 2010), Denmark and Germany (Birk Moller et al., 2007), Czech Republic (Kozak et al., 2006), and Slovakia (Polak et al., 2013). Although a relatively high number of alleles with large deletions were supposedly a characteristic of Slavic (Czech, Polish, Slovak) PKU populations (Bik‐Multanowski & Pietrzyk, 2008; Kozak et al., 2006; Polak et al., 2013), deletion of exon 5 reached a relative frequency of 14.9% in Chile (Hamilton et al., 2017). Moreover, the relative frequency reported by Kozak et al. (2006) in the Czech Republic (31 large deletions among 1042 mutant alleles) did not differ significantly from ours (three large deletions among 204 mutant alleles ‐ *p *=* *.2313), and both were significantly higher than the frequency in the Germanic population of Birk Moller et al. (2007) [four among 1,140 alleles ‐ *p *<* *.0001 and *p *=* *.0362, respectively]. As MLPA was not used in the investigation of Mexican and Portuguese PKU alleles, the frequency of large deletions in these populations cannot be estimated from traditional DNA analysis results.

Kozak et al. (2006) observed, in the Czech Republic, that a 955 bp deletion covering exon 5 (from intron 4 to intron 5; g.50448_51402del955) was associated with haplotype 1.7 and that a longer 4232 bp deletion of exon 5, with an insertion of 268 bp in the breakpoint junction (g.47563_51794del4232 g.56161_56430ins268), was associated with haplotype 4.3. Calì et al., in Italy, confirmed that g.50448_51402del955 was associated with VNTR 7. Our three c.442‐?_509+?del mutant alleles were associated with VNTR 8, two on haplotype 14/15, and the third on a non‐determined haplotype. We plan to determine the breakpoints of these three mutant alleles in the future.

Concluding, the Iberian Peninsula, especially Portugal, is the major source of PKU alleles in Rio de Janeiro. This does not mean that our PKU patients are predominantly of European descent. Although ethnic origin was not thoroughly investigated, many patients were blacks or mestizos, some of which carried rare mutations, for example, c.498C>G (p.Y166*), or mutations on new haplotypes, for example, c.250G>T (p.D84Y) on 15.9, and c.526C>T (p.R176*) on 7.8. We could not find massive independent genetic incidents as the one that occurred in Mexico, where the c.60+5G>T variant, a very rare pathogenic variant in Spain, was identified in 20.8% of the alleles (Vela‐Amieva et al., 2015) (Figure 4c). Nevertheless, the presence of a novel variant, c.934G>T (p.G312C), and of rare mutations previously described in geographical areas that had a very scanty contribution to Rio de Janeiro's population gene pool, like China, prove that autochthonous events, besides genetic drift and founder effect, played important roles in shaping Rio de Janeiro's *PAH* mutational spectrum.

## DISCLOSURE OF POTENTIAL CONFLICTS OF INTEREST

Dr. Vieira Neto reports a public grant from Coordination for the Improvement of Higher Level Personnel (Capes) of the Ministry of Education, Brazil, and private grants from FBM Pharmaceutical Industry Ltd., Anápolis, Goiás, Brazil, and from Danone Ltd., São Paulo, Brazil, during the conduct of the study.
