# Supplementary material for: Mutation analysis of the PAH gene in phenylketonuria patients from Rio de Janeiro, Southeast Brazil
Source: Mol Genet Genomic Med. 2018 May 10;6(4):575–91. doi: 10.1002/mgg3.408 (PMC6081236; doi:10.1002/mgg3.408)
Supplement: Supplementary file 1 [file MGG3-6-575-s001.docx]

Supplementary Table S1. PCR amplification primers for the 13 exons and intronic boundaries of the *PAH* gene used in this study

| **Name** | **Sequence** | **Tm (^o^C)** | **% G/C** | **Base count** | **Expected product size** |
| --- | --- | --- | --- | --- | --- |
| PAH_ex1-F | tgtaaaacgacggccagtTTCACGTGCCCTCTAGCTGT | 61.19 | 55.0 | 38 | 761 |
| PAH_ex1-R | caggaaacagctatgaccCCAGCAGTCTTCGGATCTCT | 58.89 | 55.0 | 38 |  |
| PAH_ex2-F | tgtaaaacgacggccagtCTTGCTTTGTCCATGGAGGT | 58.08 | 50.0 | 38 | 527 |
| PAH_ex2-R | caggaaacagctatgaccGTTAATCATGCCTAATCACACA | 54.53 | 36.4 | 40 |  |
| PAH_ex3-F | tgtaaaacgacggccagtATGTGAACTAACTGCCCCACC | 60.27 | 52.4 | 39 | 630 |
| PAH_ex3-R | caggaaacagctatgaccTTAATCCCCCAAACAGTCTTCC | 57.96 | 45.5 | 40 |  |
| PAH_ex4-F | tgtaaaacgacggccagtGGTGGGAGGGAGATGAGTTTT | 59.36 | 52.4 | 39 | 696 |
| PAH_ex4-R | caggaaacagctatgaccACCTCCATAGATGTACACAGGC | 59.30 | 50.0 | 40 |  |
| PAH_ex5-F | tgtaaaacgacggccagtGAGAGCCCCCATTCAAAGCAT | 60.69 | 52.4 | 39 | 244 |
| PAH_ex5-R | caggaaacagctatgaccAAGCAGGCTAGGGGTGTGTT | 61.43 | 55.0 | 38 |  |
| PAH_ex6-F | tgtaaaacgacggccagtCCCTCTGCTAACCTAACCTGC | 60.13 | 57.1 | 39 | 549 |
| PAH_ex6-R | caggaaacagctatgaccTACACGGCAAAATCCACAGC | 59.12 | 50.0 | 38 |  |
| PAH_ex7-F | tgtaaaacgacggccagtACCTAAAGGTCTCCTAGTGCCT | 59.96 | 50.0 | 40 | 303 |
| PAH_ex7-R | caggaaacagctatgaccAGCAGGAAAAGATGGCGCTC | 61.03 | 55.0 | 38 |  |
| PAH_ex8-F | tgtaaaacgacggccagtGGGAGAGGGATCATAAGCCT | 57.96 | 55.0 | 38 | 392 |
| PAH_ex8-R | caggaaacagctatgaccTAGCTTCCCAGAACCACACAC | 59.93 | 52.4 | 39 |  |
| PAH_ex9-F | tgtaaaacgacggccagtCTCCTGTGATGTTTCCAGGGTA | 59.43 | 50.0 | 40 | 959 |
| PAH_ex9-R | caggaaacagctatgaccCCAGTGGACAAGTGTCATCAGA | 59.96 | 50.0 | 40 |  |
| PAH_ex10-F | tgtaaaacgacggccagtTCCACTGACTCACATGCCAAT | 59.65 | 47.6 | 39 | 472 |
| PAH_ex10-R | caggaaacagctatgaccTCTGTAAAACCCACAGCCATCA | 59.89 | 45.5 | 40 |  |
| PAH_ex11-F | tgtaaaacgacggccagtAGAAGGAATCGGGGTGAGATG | 59.23 | 52.4 | 39 | 539 |
| PAH_ex11-R | caggaaacagctatgaccGCAGGAGGGTTCAGTGTCTTG | 60.88 | 57.1 | 39 |  |
| PAH_ex12-F | tgtaaaacgacggccagtTCTTGGAGCCAGGGGACTAA | 59.88 | 55.0 | 38 | 472 |
| PAH_ex12-R | caggaaacagctatgaccGGCGATGGTAGGGAAAGACA | 59.46 | 55.0 | 38 |  |
| PAH_ex13-F | tgtaaaacgacggccagtGCACCCAGCTCATCCAAGAAG | 61.29 | 57.1 | 39 | 514 |
| PAH_ex13-R | caggaaacagctatgaccAGCTTGAATGAAGCAGGTCCCA | 62.22 | 50.0 | 40 |  |
